# The Possible Potential Therapeutic Targets for Drug Induced Gingival Overgrowth

**DOI:** 10.1155/2013/639468

**Published:** 2013-04-16

**Authors:** Tamilselvan Subramani, Vidhya Rathnavelu, Noorjahan Banu Alitheen

**Affiliations:** ^1^Department of Cell and Molecular Biology, Faculty of Biotechnology and Biomolecular Sciences, University Putra Malaysia, 43400 Serdang, Selangor, Malaysia; ^2^Department of Oral and Maxillofacial Pathology, Faculty of Dental Science, Sri Ramachandra University, Porur, Chennai 600116, India

## Abstract

Gingival overgrowth is a side effect of certain medications. The most fibrotic drug-induced lesions develop in response to therapy with phenytoin, the least fibrotic lesions are caused by cyclosporin A, and the intermediate fibrosis occurs in nifedipine-induced gingival overgrowth. Fibrosis is one of the largest groups of diseases for which there is no therapy but is believed to occur because of a persistent tissue repair program. During connective tissue repair, activated gingival fibroblasts synthesize and remodel newly created extracellular matrix. Proteins such as transforming growth factor (TGF), endothelin-1 (ET-1), angiotensin II (Ang II), connective tissue growth factor (CCN2/CTGF), insulin-like growth factor (IGF), and platelet-derived growth factor (PDGF) appear to act in a network that contributes to the development of gingival fibrosis. Since inflammation is the prerequisite for gingival overgrowth, mast cells and its protease enzymes also play a vital role in the pathogenesis of gingival fibrosis. Drugs targeting these proteins are currently under consideration as antifibrotic treatments. This review summarizes recent observations concerning the contribution of TGF-**β**, CTGF, IGF, PDGF, ET-1, Ang II, and mast cell chymase and tryptase enzymes to fibroblast activation in gingival fibrosis and the potential utility of agents blocking these proteins in affecting the outcome of drug-induced gingival overgrowth.

## 1. Introduction

**Figure 1:**
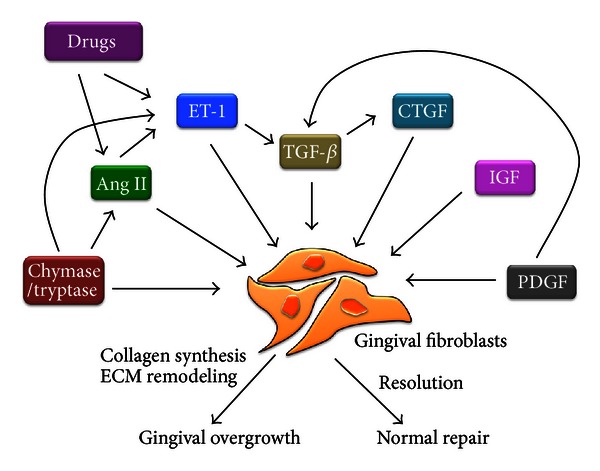
Schematic diagram of interplay among profibrotic mediators. These proteins promote fibroblast activation and gingival fibrosis. Ang II: angiotensin II; CTGF: connective tissue growth factor; ET: endothelin; IGF: insulin-like growth factor; PDGF: platelet-derived growth factor; TGF: transforming growth factor; ECM: extracellular matrix.

Drug-induced gingival overgrowth is an adverse drug reaction mainly described with three types of commonly prescribed drugs, namely, calcium channel blockers (nifedipine, diltiazem, and verapamil) [1–4], antiepileptic drugs (phenytoin) [5] and, immunosuppressants (cyclosporine) [6]. Drug-induced gingival overgrowth usually occurs within the first 3 months of starting the medication and begins as an enlargement of the interdental papilla [3]. As mentioned elsewhere, not all the patients taking these drugs develop drug-induced gingival overgrowth. While the incidence of this side effect can be as high as 65% in epileptics, 70% in transplant patients, and 30% in hypertension subjects, variation exists in the reported prevalence and severity of the clinical problem [7–10]. The degrees of inflammation, fibrosis, and cellularity depend on the duration, dose, and identity of the drug and on the quality of oral hygiene. Evidence suggests that genetic factors also might have a significant role in the pathogenesis of drug-induced gingival overgrowth and in the patient's susceptibility to this unwanted effect. A genetic predisposition could influence a variety of factors in the drug-plaque-induced inflammation. These include gingival fibroblast functional heterogeneity, collagenolytic activity, drug metabolism, and collagen synthesis [7, 11]. The effective management of these patients clearly requires the active involvement of both dental and medical professionals to minimize the possibility of complications. The rationally designed antifibrotic therapies are likely to be invaluable in curbing this problem largely because the underlying basis of fibrosis is unclear. However, numerous studies conducted over three decades have suggested that chronic inappropriate increases in levels of local and circulating chemical mediators such as angiotensin II (Ang II) and endothelin-1 (ET-1) and fibrogenic cytokines/proteins such as transforming growth factor (TGF), connective tissue growth factor (CTGF), insulin-like growth factor (IGF), platelet-derived growth factor (PDGF), and mast cell chymase and tryptase enzymes are likely to be key driving forces culminating in gingival overgrowth (Figure 1). Collectively, these hormones and cytokines result in the activation of gingival fibroblasts cells within connective tissue. Although gingival fibroblasts are induced in response to normal tissue injury, these cells disappear, thereafter, probably by apoptosis. In DIGO, however, gingival fibroblasts persist, resulting in the excessive production and remodeling of extracellular matrix (ECM). Some studies demonstrated that drugs such as cyclosporine A are able to inhibit production of ECM by gingival fibroblast and cell proliferation *in vitro* [12]. In contrast, others showed that the accumulation of proteins in ECM, particularly collagen, may occur due to an imbalance between the synthesis and the degradation of ECM, being the possible cause of the drug-induced gingival overgrowth [13]. These results suggested the imbalance in the ECM synthesis and degradation that leads to drug-induced gingival overgrowth. The mechanisms that trigger drug-induced gingival overgrowth have not been completely understood, and, although the literature data are extensive, they are quite contradictory. Recent evidence has suggested that differentiation of resident fibroblasts occurs in response to above-mentioned mediators, which are all likely to play key roles in this process. In this review, we focused on growth factors such as, TGF-*β*, CTGF, IGF, and PDGF; mast cell enzymes chymase and tryptase; circulating mediators Ang II and ET-1, and discussed their roles in the pathogenesis of drug-induced gingival overgrowth.

## 2. Growth Factors

### 2.1. Transforming Growth Factor-*β*

TGF-*β* expression is elevated in response to injury [14]. There is an extensive literature that discusses the basics of TGF-*β* signaling and its relationship to fibrosis [15–17]. Briefly, there are 3 TGF-*β* isoforms, namely, TGF-*β* 1, TGF-*β* 2, and TGF-*β* 3. These are initially present within a complex containing latent TGF-*β*-binding proteins that are proteolytically removed to release active TGF-*β*. Substantial evidence supports a central role for TGF-*β* in fibroblast activation. When applied to fibroblasts, TGF-*β* directly induces ECM gene expression and promotes ECM deposition by simultaneously suppressing matrix metalloproteinase gene expression and inducing tissue inhibitors of matrix metalloproteinase gene expression [18–20]. It is well established that the immunosuppressive drug, cyclosporine, upregulates TGF-*β* 1 synthesis, a fact that might explain the fibrogenic effect of this drug in a variety of cells and tissues [21–26]. Significantly, *in vivo* studies in animal models have shown that cyclosporine increases kidney TGF-*β* 1 levels [27]. Evidence from the study of isolated gingival fibroblasts in culture demonstrates that these cells exhibit a proliferative response to TGF-*β* 1 [25–29]. Furthermore, hyper-responsiveness of gingival fibroblasts to autocrine effects of TGF-*β* 1, coupled with increased levels of synthesis in these cells, may be important pathogenic elements in hereditary gingival fibromatosis, a condition that has histopathological features in common with drug-induced gingival overgrowth [30–32]. TGF-*β* 1 has an important profibrogenic role, not only by inhibiting the synthesis of metalloproteinases but also by stimulating synthesis of collagen [33]. In fibroblasts TGF-*β* generally is signaled through TGF-*β* type I receptor (Activin-Linked kinase 5) [16]. Activin-Linked kinase 5 (ALK5) inhibitors have been considered as potential antifibrotic compounds, although these small molecules have not yet been thoroughly investigated in gingival fibroblasts. These results suggest that blocking ALK5 may be useful in blocking the profibrotic effects of TGF-*β* in gingival fibroblasts. In gingival fibroblasts cells, ALK5 inhibition reduces profibrotic effect of TGF-*β* [34]. In addition to ALK5 inhibitors, anti-TGF-*β* antibodies have also been under consideration as potential antifibrotic agents [16]. All these findings suggest that broad targeting of TGF-*β* ligand might not be a viable antifibrotic strategy, but ALK5 inhibition may be a useful approach.

### 2.2. Insulin-Like Growth Factor

Insulin-like-growth-factor (IGFs-) I and II are well known to play essential roles in significant biological activities such as proliferation, differentiation, apoptosis, and adaptation in various kinds of tissues [35]. The actions of IGFs are mainly mediated via IGF receptor 1 (IGFR-1) and are modulated by six binding proteins (IGFBPs), designated as IGFBP-1 to IGFBP-6 [35, 36]. Unlike most of the other binding proteins, which act as competitive inhibitors of IGF-I receptor, IGFBP-5 acts to enhance IGF-I actions. IGF-I increases the synthesis of both IGFBP-5 and collagen [37]. Moreover, IGF-I's biological activity on fibroblasts includes stimulation of collagen production and downregulation of collagenase production, suggesting that IGF-I may be an important mediator in the development of gingival fibrosis through IGFBP-5 [38]. The greater expression of IGFBP-5 in periodontal ligament fibroblast (PDLF) together with IGF-I induced reduction of apoptosis in PDLF suggests a potential role of IGFBP-5 in the upregulation of IGF-I pathway [38]. Furthermore, augmented expressions of IGFBP-5 were identified in cyclosporine-induced gingival overgrowth and associated with increased collagen and fibrosis [39]. It also demonstrated that, in rat gingival cells, the cyclosporine triggers IGF-I induced proliferation and extracellular matrix synthesis [40]. IGF-I receptor inhibitors have been considered as potential target, and IGF-I receptor antagonist (A12) administration decreased lung fibrosis in bleomycin-treated mice [41]. IGF-I receptor antagonist has been identified as possible new therapeutic approaches in kidney, lung, and cardiac fibrosis, but it has not yet been investigated thoroughly in gingival fibrosis. 

### 2.3. Platelet-Derived Growth Factor

PDGF comprises a family of homo- or heterodimeric growth factors including PDGF-AA, PDGF-AB, PDGF-BB, PDGF-CC, and PDGF-DD. There are 2 different PDGF receptors, *α* and *β* [42]. PDGF causes neutrophils, macrophages, fibroblasts, and smooth muscle cells proliferation and migration [43, 44]. PDGF-B expression has been shown to occur early in gingival wound healing [45] and is increased in cyclosporine treatment [46]. The PDGF-BB isoform is also known to cause proliferation of periodontal fibroblasts and modulate their production of extracellular matrix components [47]. It has been demonstrated that many cell types within the overgrown gingival tissues express PDGF-A, PDGF-B, and both PDGF-receptors and are in agreement with the fact that PDGF is produced by a variety of cell types [48, 49]. Both phenytoin and cyclosporine cause increased production of PDGF AB/BB by macrophages *in vitro* [48, 50]. Furthermore, limited analysis of both control and cyclosporine-induced overgrowth tissues by immunohistochemistry and *in situ* hybridisation revealed that PDGF-B expression was restricted to some of the CD51-positive macrophages within the papillary lamina propria [49, 51]. Taken together these findings support a role for PDGF in the pathogenesis of gingival overgrowth. It has also been shown that injection of anti-PDGF-B antibodies reduces liver fibrosis [52], kidney fibrosis [53], and atrial fibrosis [54]. Although there was no specific published data available on gingival fibrosis, there is substantial evidence strongly suggesting that PGDF may be a good target for antifibrotic therapy in drug-induced gingival overgrowth.

### 2.4. Connective Tissue Growth Factor

CTGF or CCN2, is a 38 kDa secreted protein belonging to the CCN family of growth factors [55, 56]. CCN2/CTGF has been shown to promote the synthesis of various constituents of the extracellular matrix [56, 57], and its overexpression is associated with the onset and progression of fibrosis in human gingiva [58, 59]. CCN2 promotes cell adhesion and enhances adhesive signaling in response to extracellular ligands. CCN2 is an excellent surrogate marker for activated fibroblasts in fibrosis; for example, in the process of gingival overgrowth, connective tissue growth factor (CTGF/CCN2) is significantly induced in gingival fibroblasts [58]. Furthermore, CTGF/CCN2 is expressed in gingival epithelial cells *in vivo* in fibrotic tissues but not in normal tissues [59]. CTGF is rapidly and potently induced by TGF-*β* 1 in fibroblastic cells from a variety of different tissues and contributes to the regulation of extracellular matrix genes [60]. However, on its own, CCN2 is considered to only weakly promote fibrosis; rather, what CCN2 appears to do is to create an environment favorable for fibrogenic stimuli to act [61, 62]. Levels of connective tissue growth factor (CTGF/CCN2) are highest in gingival tissues from phenytoin-induced lesions, intermediate in nifedipine-induced lesions, and nearly absent from cyclosporine-A-induced overgrowth. CTGF/CCN2 levels correlate positively with fibrosis, consistent with the role of CTGF/CCN2 in promoting and maintaining fibrosis [58]. CTGF expression is related to the degree of fibrosis, as cyclosporine tissues show little CTGF expression and were demonstrated to be highly inflamed and not fibrotic.

Drugs targeting the action of CCN2, such as small interfering RNAs or neutralizing antibodies, are currently under development [63]. A CCN2 response element exists in the COL1A2 promoter, and recently it has been shown that blocking CCN2 action using an anti-CCN2 antibody or small interfering RNA reduces aspects of bleomycin-induced lung fibrosis [64]. Overall, strong *in vivo* evidence directly supporting the notion of anti-CCN2 therapies in pathologies of gingival over growth is lacking. However, it is possible that CCN2 may be a key selective modulator in the gingival overgrowth, operating downstream and in concert with TGF-*β*.

## 3. Mast Cell Proteases

Mast cells are derived from precursor cells in the bone marrow and mature under the influence of the c-kit ligand, stem cell factor with their final phenotype being dependent on the microenvironment in which they reside. While mast cells throughout the body are involved in the pathogenesis of many diseases, the role of gingival mast cells in gingival overgrowth has been understudied despite circumstantial evidence indicating their potential involvement. In addition to the storage of histamines and proteases such as tryptase and chymase in their secretory granules, gingival mast cells also produce a wide variety of cytokines, growth factors, and other biologically active mediators that are capable of mediating tissue remodeling. Several *in vitro* studies have pointed out the potential role of mast cells inducing gingival fibroblast proliferation and collagen synthesis [65]. A recently published study demonstrated that mast cell tryptase and chymase enzymes were expressed significantly higher in DIGO tissues compared to healthy gingiva and may contribute to alterations in basement membranes [66]. In addition, tryptase can interact with a number of cell types and has been shown to be mitogenic for fibroblasts [67] and epithelial cells [68]. The immunohistochemical expression of mast cell tryptase in inflammatory fibrous gingival hyperplasia demonstrated the involvement of mast cells in the induction of fibrosis by observing increased mast cell degranulation in fibrous tissues [69]. Mast cell chymase-positive gingival overgrowth patients showed significantly increased fibrotic lesions in the gingival tissues compared with chymase-negative patients [70]. 

Ang II, which is generated through angiotensin-converting enzyme (ACE), stimulates growth of the gingival tissue. However, an alternative generation pathway, chymase, also exists in the gingiva, and chymase has a higher specificity for the conversion of Ang I to Ang II. In addition, mast cells store latent transforming-growth-factor-(TGF-) *β* in an intracellular granule and secrete TGF-*β*, which is activated by chymase [71]. Locally synthesized chymase seems to promote gingival remodeling via the formation of Ang II and proliferative properties. Moreover, mast cells are likely to contribute indirectly to the formation of ET-1 by releasing chymase, which can generate ET-1, by cleavage of big ET-1 [72]. ET-1 is a powerful vasoconstrictor with mitogenic activity on gingival fibroblasts and facilitates gingival fibrosis [73]. Thus, the role of mast cells in the generation of fibrosis involves the direct effect of mast cell-derived ET-1 and Ang II, the exacerbation of preexisting inflammatory mediators, and the release of mast cell enzymes, such as tryptase and chymase.

The chymase and tryptase inhibitors have been considered as potential antifibrotic compounds although these inhibitors have not yet been thoroughly investigated in drug-induced gingival overgrowth. In skin mast cells, the selective inhibitor of chymase ZIGPFM inhibited histamine release and proliferation; moreover, with chymostatin, which is also a potent inhibitor of chymase, inhibition of 70 or 80% of mast cell mediators was achieved [74]. It has been demonstrated that in cardiac fibrosis the chronic chymase inhibition prevented cardiac remodeling and fibrosis [75, 76]. The similar degree of inhibition was observed with tryptase inhibitor APC366 and with certain other inhibitors of tryptic proteinases [77]. These results suggest that inhibitors of chymase and tryptase can be potent stabilizers of human mast cells and particularly those in relation to overgrown gingival tissues. It is possible that the development of potent and selective chymase/tryptase inhibitors will lead to useful new drugs for the treatment of mast cell-mediated drug-induced gingival overgrowth. 

## 4. Angiotensin II

**Figure 2:**
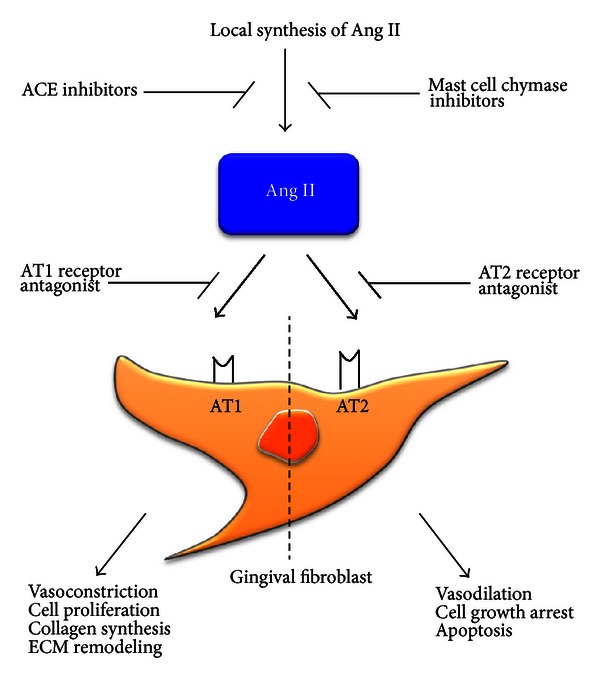
Potential role of angiotensin II and its receptors expressed by fibroblast in gingival fibrosis. Ang II: angiotensin II; AT: angiotensin receptor; ACE: angiotensin-converting enzyme; ECM: extracellular matrix.

Elevated intragingival Ang II production is found in drug-induced gingival overgrowth [78]. Angiotensin is an oligopeptide that causes vasoconstriction and increased blood pressure. Although numerous factors have been implicated in the pathogenesis of drug-induced gingival overgrowth, several lines of evidence strongly suggest the involvement of Ang II in the development of DIGO [78] (Figure 2). Ohuchi et al. [72] have reported that Ang II generated by gingival fibroblasts in response to phenytoin and nifedipine may contribute to the development of drug-induced gingival overgrowth. Recent studies indicate that Ang II and TGF-*β* 1 do not act independently from one another but act as part of an integrated signaling network that promotes cardiac remodeling and possibly fibrosis. Ang II upregulates TGF-*β* 1 expression through the angiotensin type 1 (AT1) receptor in gingival fibroblasts [79]. In an important study, it was shown that Ang II was not able to induce fibrosis *in vivo* in the absence of TGF-*β* [80]. Providing further support for this notion, Ang II induces collagen in gingival fibroblasts through TGF-*β* and ERK [81, 82]. Ang II and TGF-*β* 1 appear to act in an autocrine loop, as TGF-*β* 1 can directly stimulate AT1 receptor expression through ALK5 and Smads 2/3/4, providing a further indication of crosstalk between the TGF-*β* and angiotensin pathways [83]. These results indicate that Ang II and TGF-*β* pathways are likely to cooperate to drive fibrogenic responses *in vivo.* Drugs that inhibit the angiotensin pathway, namely, angiotensin-converting enzyme inhibitors and angiotensin receptor antagonists, are widely used to treat various fibroses. Angiotensin receptor inhibitors such as losartan appear to be effective in reducing gingival fibrosis [78]. Hilgers and Mann [84] demonstrated that treatment with an ACE inhibitor or AT1 receptor antagonist abrogates drug-induced hypertrophy. Further clinical studies showed that treatment with ACE inhibitor or AT1 receptor antagonist reduced fibrogenesis in CsA-treated patients after renal transplantation [85, 86]. Thus, compared to generally antagonizing TGF-*β* signaling, angiotensin-converting enzyme inhibitors and angiotensin receptor antagonists may be useful approaches to control gingival overgrowth.

## 5. Endothelin-1

**Figure 3:**
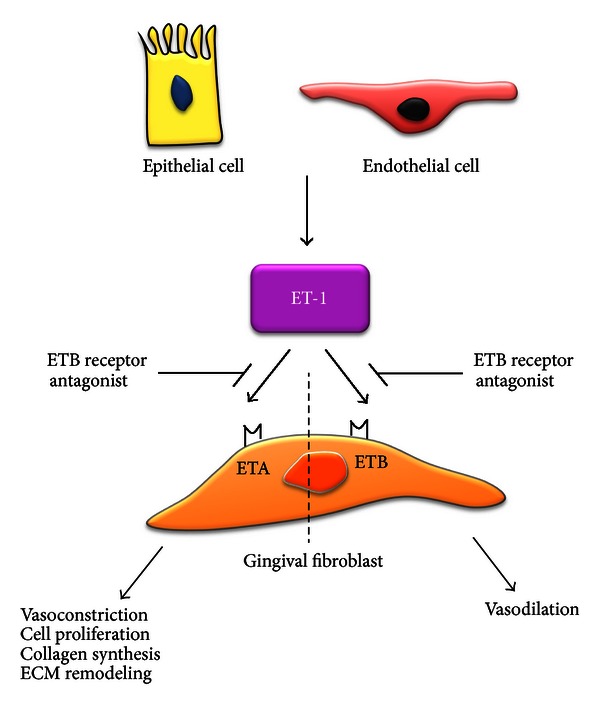
Potential role of endothelin-1 and its receptors expressed by fibroblast in gingival fibrosis. ET-1: endothelin-1; ETA: endothelin receptor A; ETB: endothelin receptor B; ECM: extracellular matrix.

Endothelin is a protein secreted from endothelial cells, and it is a powerful vasoconstrictor with mitogenic or comitogenic properties, which acts through the stimulation of 2 subtypes of receptors (ETA and ETB) [87, 88]. ET-1 is first produced in the form of a 212-aa precursor (prepro-ET-1) and then cleaved twice to form a biologically active 21-aa peptide, the last cleavage mediated by ET-converting enzyme [88]. Studies have shown the expression of ET-1 in inflamed gingival tissues and threefold increase in drug-induced gingival overgrowth tissues [72] Teder and Noble, [88] also found that ET-1 was localized in inflamed gingival epithelial cells. Similar results found that ET-1 was increased in cardiac, renal, and gingival fibrosis [89–91]. The increased ET-1 can modulate the synthesis of TGF-*β* and TNF-*α*, which are important in mediating fibroses. Our previous study demonstrated the augmented expression of TNF-*α* in drug-induced gingival overgrowth tissues compared to healthy gingival tissues [92]. ET-1 is also profibrotic by stimulating fibroblast replication, migration, and contraction, increasing fibronectin, collagen synthesis, and decreasing collagen degradation [93–96]. All of these processes have been proposed for the pathogenesis of DIGO (Figure 3). Ohuchi et al. [72] showed that phenytoin- and nifedipine-induced gingival fibroblasts proliferation was mediated through the induction of Ang II and ET-1 and probably mediated through Ang II receptor-1 and ETA in gingival fibroblasts. Some studies have suggested that TGF-*β* works together with ET-1 to promote myofibroblast differentiation [97]. TGF-*β* induces ET-1 via JNK, and ET-1 is a downstream mediator of the fibrogenic responses of TGF-*β* in normal fibroblasts [97]. Intriguingly, Ang II also induces ET-1 via ERK and reactive oxygen species [98]. These results suggest that ET operates downstream of the TGF-*β*/Ang II system to drive fibroblast activation and fibrosis.

ET receptor antagonism might be considered as an appropriate therapy for the fibrosis. Recent data demonstrate the fundamental role of endothelin in the pathogenesis of fibrosis and the antifibrotic potential of dual endothelin receptor antagonists such as bosentan [87]. It is an orally active, dual endothelin receptor antagonist, which competitively antagonizes the binding of endothelin to both endothelin receptors ETA and ETB, and prevents endothelin-induced fibroblast proliferation and extracellular matrix deposition and contraction, and reduces fibrosis [87]. Bosentan has already been approved by the Food and Drug Administration for the treatment of pulmonary arterial hypertension [99, 100]. Furthermore, macitentan is an orally active, nonpeptide dual endothelin ETA and ETB receptor antagonists for the potential treatment of idiopathic pulmonary fibrosis and pulmonary arterial hypertension [101]. Since it has the ability to target the tissues and to block both ETA and ETB receptors, macitentan is emerging as a new agent to treat cardiovascular disorders associated with chronic tissue ET system activation [101]. It is interesting to note that dual-acting angiotensin II and ET receptor blockers have been shown to reduce systemic blood pressure in animal models and in hypertensive patients [102, 103]. Preliminary data in smaller human studies have shown that these agents are safe and well tolerated [102, 103]. Thus combination ET/Ang II therapies have promise in controlling gingival overgrowth.

## 6. Conclusion

Drugs that modulate TGF-*β*, PDGF, CTGF, IGF, mast cell chymase/tryptase, Ang II, and ET-1 are being considered for protective gingival overgrowth effects and can ameliorate fibrosis. Even though these proteins are likely to cooperate in driving tissue remodeling and fibrogenic responses in fibroblasts, each protein also has certain unique features, providing a rationale that therapies targeting individual molecules might be useful. Current strategies in clinical practice combine treatments with these inhibitors and receptor blockers, due to their potential additive beneficial effects. The data presented here highlight potential interesting candidates for antifibrotic treatments. Future studies are necessary to evaluate their potential beneficial effects fully in gingival overgrowth.
